# Ultra-high-pressure liquid chromatography tandem mass spectrometry method for the determination of 9 organophosphate flame retardants in water samples

**DOI:** 10.1016/j.mex.2016.04.006

**Published:** 2016-04-20

**Authors:** María Lorenzo, Julián Campo, Yolanda Picó

**Affiliations:** a Food and Environmental Safety Research Group (SAMA), Desertification Research Centre (CIDE-UV, GV, CSIC), Faculty of Pharmacy, University of Valencia, Burjassot, Valencia, Spain; b Environmental Forensic and Landscape Chemistry Research Group, Desertification Research Centre—CIDE (Spanish Council for Scientific Research, University of Valencia, Generalitat Valenciana), Carretera Moncada— Náquera km 4.5 (Campus IVIA), 46113 Moncada, Valencia, Spain; c Earth Surface Science, Institute for Biodiversity and Ecosystems Dynamics, University of Amsterdam, Science Park 904, 1098 XH Amsterdam, The Netherlands

**Keywords:** Analysis of organophosphate flame retardants in water by SPE and UHPLC–MS/MS using a trap column., Organophosphate flame retardants, Interferences, Trap column, LC–MS/MS, Water, solid phase extraction

## Abstract

Few methods are available for comprehensive organophosphate flame retardants (PFRs) detection in water and wastewater. Gas chromatography has been employed previously, but this approach is less selective, not amenable for use with deuterated standards and can suffer unfavorable fragmentation. Ultra-high-pressure liquid chromatography tandem mass spectrometry (UHPLC-QqQ-MS/MS) has become the most promising platform, already applied successfully for analysis of selected PFRs in some environmental matrices like water and wastewater. However, the presence of some interferences from the dissolvent, the equipment and the used materials should be taken into account. The procedure involves:

• The first determination of PFRs by UHPLC-QqQ-MS/MS using a trap column to distinguish the interferences coming from the instrument and mobile phases.
• The optimization of the LC separation to distinguish all target compounds and their interferences.
• This method coupled to a solid-phase extraction (SPE) improve the detection and quantification of PFRs.

## Graphical abstract

**Figure:**
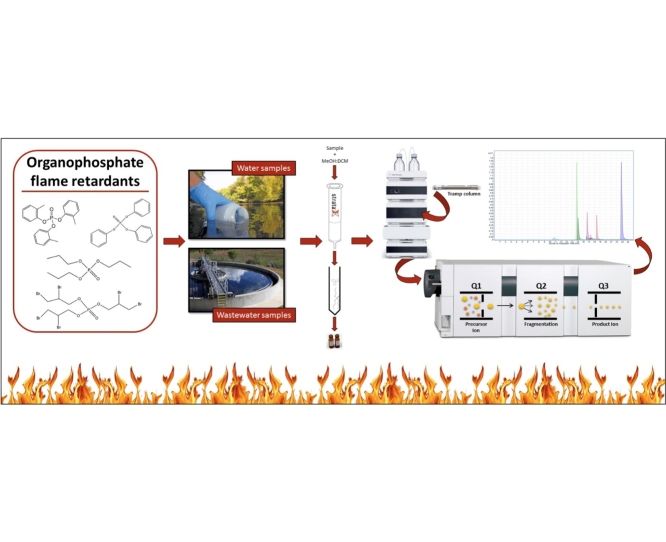

## Method details

### Interference analysis

Ultra-high-pressure liquid chromatography tandem mass spectrometry (UHPLC-QqQ-MS/MS) is likely the most promising platform for the determination of PFRs in water and wastewater matrices [1], [2], [3], [4], [5]. However, the presence of some interferences from the dissolvent, the equipment and the laboratory material can disturb their analytical determination. We made, up to our knowledge, the first determination of PFRs using a trap column to distinguish these interferences. Furthermore, we prove two different solvents, acetonitrile (ACN) and methanol (MeOH), observing the presence of analyte’s contamination in both cases. The system is applied to the determination of PFR in water.

**Fig. 1:**
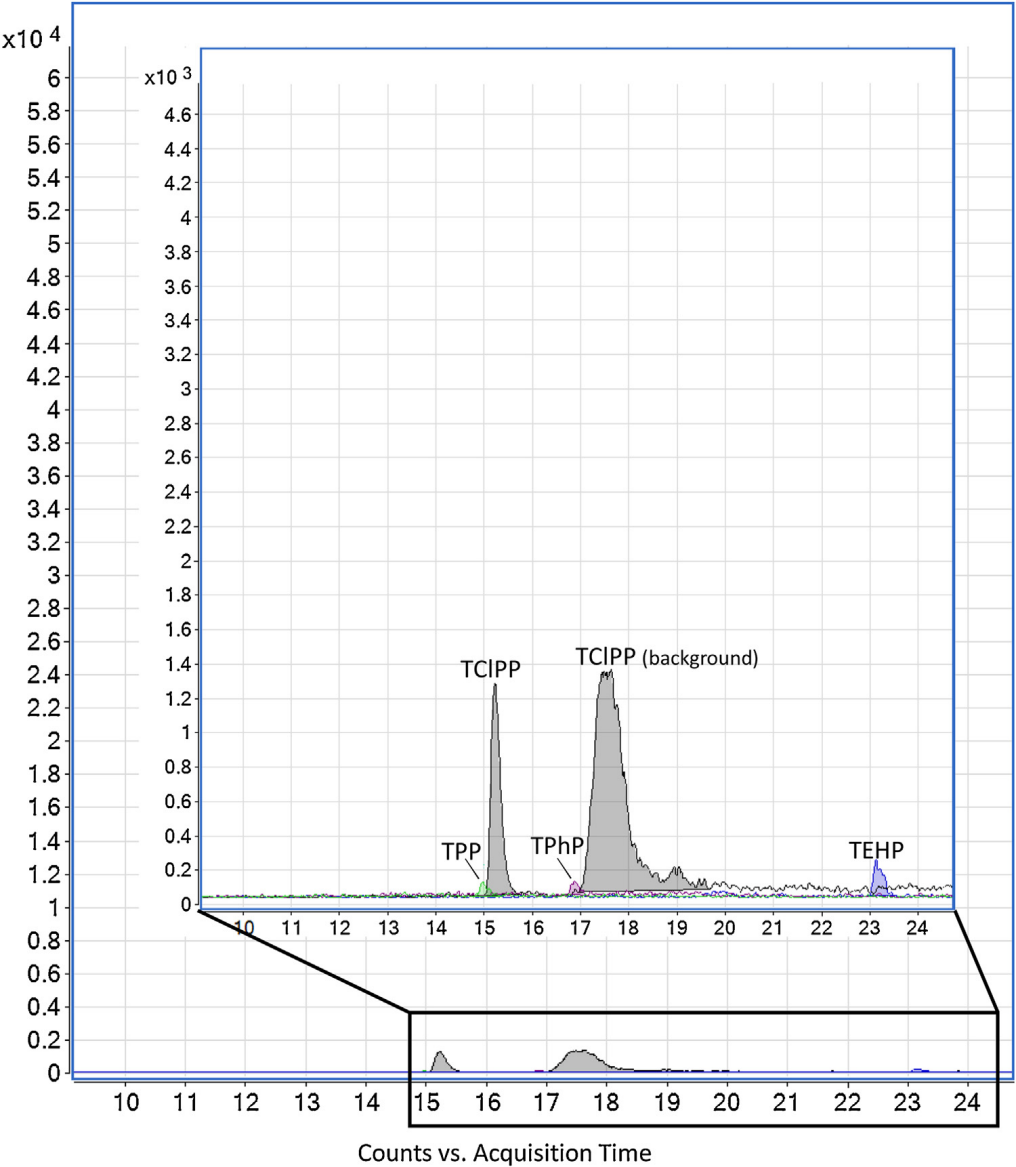
Chromatogram of a river sample.

**Fig. 2:**
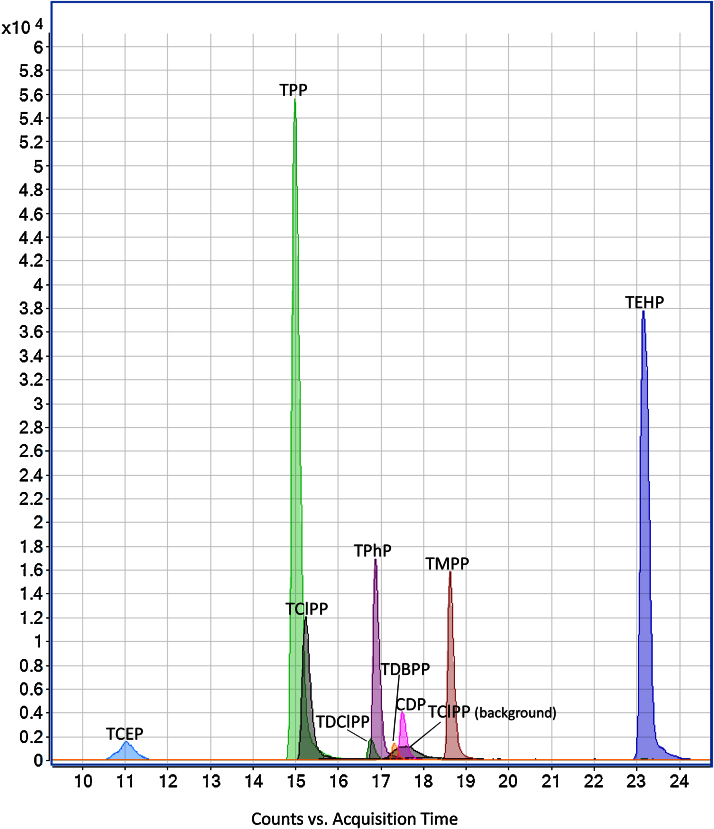
Chromatogram of the previous river sample spiked at 500 ng/mL.

Fig. 1, Fig. 2 present chromatograms of a non-spiked river sample and the same sample spiked with the analytes.

### Reagents and samples

(a) **Compounds:** the nine PFRs and the four deuterated PFRs used as internal standard (IS) (Table 1**)** were purchased from LGC Standards (Germany).
(b) **Solvents:** methanol (MeOH), dichloromethane (DCM) and acetonitrile (ACN) were bought from VWR (Radnor, PA, USA) and formic acid from AMRESCO (Solon, OH, USA), all of them were of analytical quality.
(c) **Ultra-pure water**: prepared with Milli-Q SP Reagent Water System from Millipore (Bedford, MA, USA).
(d) **Standard solutions**: individual solutions (1 mg/mL) were prepared by dissolving the PFRs in MeOH. Mixed stock solutions containing 10000 ng/mL and 100 ng/mL of each of the nine PFRs were prepared by dilution of the stock solutions with MeOH. Stock and mixed standard solutions were stored in polypropylene tubes at 4 °C.
(e) **Samples**: water samples were obtained from the Turia River (May 2015) from the influent and the effluent of the wastewater treatment plant (WWTP) of Pinedo (March 2015), both near the city of Valencia (Spain). Influent wastewater samples were filtered under vacuum using the ADVANTEC^®^ filters to remove the particulate matter. Analysis of spiked samples and blanks were made in triplicate.

### Optimization of sample extraction

(a) Put the Phenomenex Strata-X 33u Polymeric Reversed Phase (200 mg/6 mL) cartridges (Phenomenex, Torrance, Ca, USA) into a 12 port vacuum manifold Supelco Visiprep 57030-U de Sigma-Aldrich (St. Louis, MO, EEUU).
(b) Precondition the cartridge with 6 mL MeOH:DCM (1:1 v/v), 6 mL of MeOH and 6 mL of water, with 350 mba/h·PA vacuum.
(c) Pass the water samples through the cartridges under previous vacuum at a flow rate of 10 mL/min.
(d) Dry the cartridges under vacuum for 15 min.
(e) Elute the analytes on a 15 mL Falcon tube VWR (Radnor, PA, USA) with 10 mL of MeOH:DCM (50:50 v/v).
(f) Evaporate the extracts to dryness at 40 °C using a combined sample concentrator model SBHCONC/1 and a heating plate model SBH130D/3 both manufactured by Stuart (Stafford, UK).
(g) Redissolve the residue in 1 mL of methanol by agitation and ultrasonication for 1 min and pass the extract to 2 mL amber vials with 250 μL insert polypropylene 100/PK + Septum Sil/PTFE, both manufactured by Análisis Vínicos S.L. (Tomelloso, Spain).

### Instrumentation

The analysis was performed using:

(a) **UHPLC-QqQ-MS/MS system**: 1260 Infinity ultra-high-performance liquid chromatograph combined with a 6410 triple quadrupole mass spectrometer (MS/MS) with electrospray ionization (ESI) of Agilent Technologies (Santa Clara, CA, USA).
(b) **LC column**: Kinetex C18 (50 × 2.1 mm, 1.7 μm) from Phenomenex (Torrance, CA, EEUU).
(c) **Trap column**: Zorbax Eclipse Plus C18 (30 × 4.6 mm, 3.5 μm) from Agilent Technologies (Santa Clara, CA, USA). Place this column between the pump and the auto sampler.
(d) **Mobile phases**: (A) water and (B) methanol, both containing 0.1% of formic acid.
(e) **Gradient**: 0 min (30% B), 0.5 min (30% B), 12 min (95% B), 18 min (98% B) and 25 min (98% B) and return to the initial conditions. An equilibration time of 15 min was applied to stabilize the column conditions.
(f) **Flow rate**: 0.2 mL/min and the sample volume injected was 5 μL.
(g) **Analysis**: performed in positive ionization mode.
(h) **Data acquisition**: carried out in selected reaction monitoring (SRM) to identify and quantify using two precursor-product ion transitions, retention times, and the ratio of intensities between the two product ions.

Fragmentor and collision energies were optimized for each compound individually (Table 2).

### Validation of the method

The validation of the instrumental parameters (Table 3**)** was performed by determining recoveries, limit of detection (LOD), limit of quantification (LOQ) and linearity over the range to obtain suitable R^2^. The quantification was performed using the internal standard method. The mixture of the internal standards was added to water to a concentration of 200 ng/L to get a final concentration in the injected extract of 50 ng/mL. The reported parameters were calculated from distilled, river and wastewater samples spiked with standard solutions prepared in methanol at the appropriate concentrations, in a way that the volume of organic solvent added were never higher than 250 μL. The samples were processed with plastic materials as much as possible to avoid adsorption to glass. There were not river or wastewater samples free of PFRs. Then, to perform these experiments several non-spiked samples were analyzed and the peak area of those compounds were subtracted to that found in the spiked samples.

The LOD was calculated as the mass of analyte required to produce a signal-to-noise (S/N) ratio of 3:1 and the LOQ of 10:1. S/N ratios were calculated using MassHunter Workstation Software (GL Sciences, Tokyo, Japan). LODs and LOQs were estimated from water samples spiked at 4 ng/L for river water and at 100 ng/L in wastewater. Values reported in Table 3 are LODs and LOQs in the injected extracts that would correspond to LODs ranging from 0.12 to 1 ng/L and LOQs from 1.2 to 10 ng/L in water samples. The results obtained for the LODs and LOQs show that the proposed method is sensitive enough for the determination of the PFRs in water samples.

The precision studies were carried out from the evaluation of intra-day and inter-day variations of the areas ratio between the analyte and its IS using water extracts suitably stored. The intra-day precision of peak areas ratio (five replicates at 400 ng/L), expressed by means of the percentage of relative standard deviation (%RSD (*n* *=* *5*)) were lower than 11.4% and the inter-day precision (five replicates) were less than 20%. Recoveries were calculated from samples spiked at 400 ng/L analyzed in quintuplicate.

The calibration of the LC–MS/MS was conducted using seven different concentrations (from the LOQ to 300 ng/mL) of PFR standard solutions in methanol with 50 ng/mL of each deuterated compound used as IS. The use of IS prevent the matrix effects

The robustness of the method was clearly ascertained during the optimization procedure, by establishing the consequences of the deliberate introduction of minor reasonable variations (mostly different water volume analyzed, sample flow-rates during the extraction step, cartridge drying times and eluent composition) and by the similar results obtained checking different types of water. The results obtained from the variations of ±20% in the different parameters were not significantly different from those achieved by the validated method. These results proved that the proposed method was robust.

Each 10 samples, one instrumental and one procedural blank were analyzed to serve as quality control. Samples analyzed shown clearly two peaks, the first one corresponding to the TClPP found in the river water and the second to the instrumental background, indicating background contamination from the injection system and tubing of the LC–MS/MS can be successfully separated from the sample contamination. Table 4 shows the performance of the system in water samples (river, influent and effluent wastewater).

## Figures and Tables

**Table 1:** List of the compounds name, acronym and formula.

Compounds	Acronym	Formula	CAS number
Tris(2-chloroethyl) phosphate	TCEP	C_6_H_12_Cl_3_O_4_P	115-96-8
Tri-*n*-propylphosphate	TPP	C_9_H_21_O_4_P	513-08-6
Tris(2-chloroisopropyl)phosphate	TClPP	C_9_H_18_Cl_3_O_4_P	13674-84-5
Tris(1,3-dichloro-2-propyl)phosphate	TDClPP	C_9_H_15_Cl_6_O_4_P	13674-87-8
Triphenyl phosphate	TPhP	C_18_H_15_O_4_P	115-86-6
Cresyl diphenyl phosphate	CDP	C_19_H_17_O_4_P	26444-49-5
Tris(2,3-dibromopropyl)phosphate	TDBPP	C_9_H_15_Br_6_O_4_P	126-72-7
Tricresylphosphate	TMPP	C_21_H_21_O_4_P	1330-78-5
Tris-(2-ethylhexyl)phosphate	TEHP	C_24_H_51_O_4_P	78-42-2
Triphenyl phosphate D15 (IS)	M-TPHP	C_18_D_15_O_4_P	1173020-30-8
Tris(1,3-dichloro-2-propyl) phosphate D15 (IS)	M-TDCIPP	C_9_D_15_Cl_6_O_4_P	Not available
Tris-(2-chloroethyl) phosphate D12 (IS)	M-TCEP	C_6_D_12_Cl_3_O_4_P	115-96-8
Tris(2-chloroisopropyl) phosphate D18 (IS)	M-TCIPP	C_9_D_18_Cl_3_O_4_P	13674-84-5

**Table 2:** Dynamic MRM conditions for UHPLC-QqQ-MS/MS determination of PFRs.

Acronym	Associated IS	Precursor Ion	Product ion	Fragmentor	Collision Energy
TCEP	M-TCEP	287	99	100	15
		285	223	100	10
TPP	M-TPhP	225.1	140.9	84	3
		225.1	98.9	84	15
TClPP	M-TClPP	329	99	80	15
		327	175	80	10
TDClPP	M-TDClPP	432.9	99.1	80	15
		430.9	99.1	80	15
TPhP	M-TPhP	327.1	214.9	117	27
		327.1	151.9	117	43
CDP	M-TPhP	341.1	151.9	167	43
		341.1	90.9	167	39
TDBPP	M-TDClPP	698.6	98.9	120	25
		696.6	98.9	120	25
TMPP	M-TPhP	369.1	165.6	192	31
		369.1	91	192	43
TEHP	M-TPhP	435.4	98.9	113	7
		435.4	71	113	5
*M-TPHP*	–	342	160	120	47
		342	82	120	47
*M-TDCIPP*	–	448	102	120	15
		446	102	120	15
*M-TCEP*	–	299	67	100	20
		297	67	100	20
*M-TCIPP*	–	347	102	100	20
		345	102	100	20

In italics, the compounds used as IS.

**Table 3:** Recoveries and R^2^ values for calibration curves (n = 7), LODs and LOQs (ng/mL) and intra and inter-day precision.

Acronym	Recoveries	R^2^	LOD (ng/ml)	LOQ (ng/ml)	Intra-day precision	Inter-day precision
TCEP	98%	0.994	0.03	0.3	7.5	6.6
TPP	95%	0.990	0.25	2.5	5.8	19.6
TClPP	102%	0.990	0.25	2.5	3.0	6.6
TDClPP	96%	0.990	0.03	0.3	4.1	8.2
TPhP	96%	0.998	0.1	1	4.2	18.9
CDP	94%	0.994	0.1	1	2.6	19.9
TDBPP	99%	0.998	0.25	2.5	11.4	17.5
TMPP	101%	0.990	0.1	1	3.2	18.6
TEHP	106%	0.992	0.25	2.5	5.9	20.0

**Table 4:** Analysis of 250 mL of the river water and the influent and effluent wastewater samples (ng/ml).

Acronym	River water	Influent wastewater	Effluent wastewater
TCEP	<LOQ	19.0	0.8
TPP	4.0	<LOQ	<LOQ
TClPP	72.5	448.9	134.6
TDClPP	<LOQ	24.4	20.5
TPhP	5.6	20.2	9.7
CDP	<LOQ	298.5	23.7
TDBPP	<LOQ	393.9	7.3
TMPP	<LOQ	17.0	2.2
TEHP	12.5	9.7	99.9
