# Supplementary material for: Ultra-high-pressure liquid chromatography tandem mass spectrometry method for the determination of 9 organophosphate flame retardants in water samples
Source: MethodsX. 2016 Apr 20;3:343–9. doi: 10.1016/j.mex.2016.04.006 (PMC4865636; doi:10.1016/j.mex.2016.04.006)
Supplement: Supplementary file 1 [file mmc1.docx]

# **Additional information**

PFRs have become increasingly important in recent times because the significant reduction in the use of polybrominated diphenyl ethers (PBDEs) - persistent organic pollutants, dangerous for the environment. PFRs are a suitable alternative because they unstable and metabolized quickly in living organisms. Since several decades ago, these compounds were exploited in the production of dyes, varnishes, adhesives, synthetic resins, polyvinyl chloride, hydraulic fluids, plastics and textiles [1-3]. The occurrence, fate and metabolism of OFRs has been and is a hot spot of environmental research.

One of the most important problems associated to the analysis of PFRs in environmental matrices is contamination during all stages of the analytical procedure. Some authors have highlighted laboratory contamination (glass, plastic and rubber material, dissolvent, instruments, etc.) as an important problem in the analysis of contaminants not fully solved yet [[6-10](#_ENREF_6)]. To reduce or eliminate instrumental background contamination in the case of phthalates [[11](#_ENREF_11)] and perfluoroalkyl substances [[12](#_ENREF_12), [13](#_ENREF_13)], the UHPLC plumbing was altered inserting a trap column between the pump and the injector, this delay the elution of the compounds coming from the instruments from those coming from the sample extracts eliminating interferences and improving the accuracy of the quantification. Up to our knowledge, the insertion of a trap column to eliminate background contamination by BFRs has not been tested before. Its use allows to obtain a proper validation of the method in water samples.

Wang et al. 2011 first studied the possible blank contamination by PFRs from the sample treatment or mobile phase of the LC [2]. They found blank contamination in both, methanol and acetonitrile, but they select acetonitrile for the mobile phase because it provided much cleaner instrumental background. We also tested both solvents (methanol and acetonitrile) and also found background contamination in the two solvents (Fig. S1) but in our case, the methanol was selected because provides better separation and the interferences can be minimized using the trap column.

PFR extraction from water samples has been carried out by liquid–liquid extraction (LLE) [4] or solid-phase extraction (SPE) [2] but also by microextraction approaches such as solid-phase microextraction (SPME) [[14](#_ENREF_14)]. Nevertheless, SPE has been the most frequently selected technique because it provides robust results and high sample throughput. In our study, all the selected OFRs were determine in water samples using SPE with Oasis HLB. After elution of the SPE cartridge with methanol-dichloromethane (50:50, v/v), Analytes can be recovered in an extension higher than 94 %, without the need of adjusting the pH of the samples. The performance of the method has been evaluated using environmental water samples with different complexities.


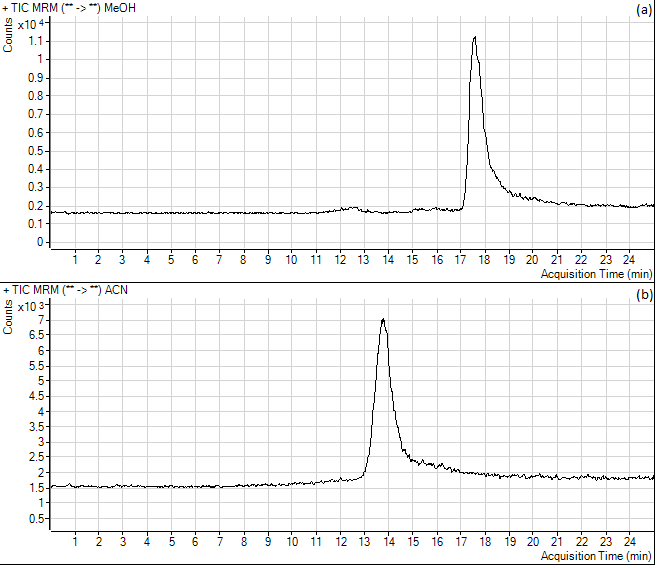


**Figure S1.** Background contamination in (a) MeOH with MeOH as mobile phase and (b) ACN with CAN as mobile phase.

References

[6] S.H. Brandsma, et al., Organophosphorus flame-retardant and plasticizer analysis, including recommendations from the first worldwide interlaboratory study, TrAC Trends Anal. Chem. 43 (2013) 217–228.

[7] D. Chen, R.J. Letcher, S. Chu, Determination of non-halogenated, chlorinated and brominated organophosphate flame retardants in herring gull eggs based on liquid chromatography–tandem quadrupole mass spectrometry, J. Chromatogr. A 1220 (2012) 169–174.

[8] S. Chu, R.J. Letcher, Determination of organophosphate flame retardants and plasticizers in lipid-rich matrices using dispersive solid-phase extraction as a sample cleanup step and ultra-high performance liquid chromatography with atmospheric pressure chemical ionization mass spectrometry, Anal. Chim. Acta 885 (2015) 183–190.

[9] J. Cristale, S. Lacorte, Development and validation of a multiresidue method for the analysis of polybrominated diphenyl ethers, new brominated and organophosphorus flame retardants in sediment, sludge and dust, J. Chromatogr. A 1305 (2013) 267–275.

[10] I. van der Veen, J. de Boer, Phosphorus flame retardants: properties, production, environmental occurrence, toxicity and analysis, Chemosphere 88 (10) (2012) 1119–1153.

[11] Ionics, Mass Spectrometry, High Throughput LC–MS/MS Assay of Phthalates. Application Note: AN 122A.

[12] J.W. Washington, et al., Analysis of perfluorinated carboxylic acids in soils II: optimization of chromatography and extraction, J. Chromatogr. A 1181 (1–2) (2008) 21–32.

[13] Y. Picó, et al., Perfluorinated compounds in food: a global perspective, Crit. Rev. Food Sci. Nutr. 51 (7) (2011) 605–625.

[14] I. Rodríguez, et al., Suitability of solid-phase microextraction for the determination of organophosphate flame retardants and plasticizers in water samples, J. Chromatogr. A 1108 (2) (2006) 158–165.
